# The effects of vacuum fluctuations on teleportation of quantum Fisher information

**DOI:** 10.1038/srep40193

**Published:** 2017-01-09

**Authors:** Yao Jin

**Affiliations:** 1 School of Electronic and Communication Engineering, Guiyang University, Guiyang, Guizhou 550005, China

## Abstract

The teleported quantum Fisher information of the phase parameter of atomic state is studied in consideration of vacuum fluctuations. Our results show that the teleported information is determined by the wavelength of the atoms as well as the distance of teleportation. When the wavelength of the atoms is much smaller than the teleportation distance, the teleported information decays with time and the decay rates are determined by the spontaneous emission rate of the atoms. However, when the wavelength of the atoms is much larger than the teleportation distance, the teleported information remains unchanged with time. The information of the phase parameter of atomic state has been absolutely transmitted.

Quantum teleportation, as a fascinating protocol predicted by quantum mechanics^1^, plays important role in quantum communication and quantum computation networks^2^,^3^. Quantum state, rather than the physical systems, can be transferred from one place to another different place by using two entangled physical systems locating in the two different places. In the past years, quantum teleportation has been studied both theoretically and experimentally^4^,^5^,^6^,^7^,^8^,^9^,^10^,^11^. As in many scenarios, we concern only the transformation of information of specific parameter of the quantum state, the idea of considering the quantum Fisher information (QFI)^12^,^13^,^14^,^15^,^16^ rather than the quantum states themselves as the information content in quantum information-processing tasks first appeared in the reference^17^ and then was generalized in refs 18,19. As a result, quantum Fisher information becomes the credibility of specific information teleportation^20^ as well as the fidelity in quantum state teleportation. However, in reality, interaction between the systems and environment can not be neglected, which may cause the degradation of the entanglement of the two initially entangled systems, thus affects the fidelity in quantum state teleportation^21^,^22^,^23^ as well as the teleported QFI in specific information teleportation^20^.

One environment which no systems can be isolated from is the vacuum that fluctuates all time in quantum sense. The interaction between vacuum fluctuations and one quantum system will cause the decoherence of the state of the system. However, the interaction between vacuum fluctuations and a pair of quantum systems will cause the indirectly correlations of the two quantum systems as well as the decoherence behavior of each quantum system, thus brings complex effects to the entanglement of the quantum systems as well as the procedure of teleportation. In this regard, we use the two-level atoms, which are in interaction with vacuum fluctuations of scalar field, as the initially entangled systems to study how the vacuum fluctuations affect the procedure of teleportation. In particular, we will study the teleported QFI in consideration of vacuum fluctuations.

## The procedure of teleportation

We consider two atoms were initially prepared in state 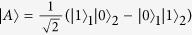. Here |1〉_1,2_ and |0〉_1,2_ are the excited and ground state of the atoms. The atom one was sent to position *P(x*_0_, *y*_0_, *z*_0_) and the atom two was sent to position *Q(x*_0_, *y*_0_, *z*_0_ + *L*). So the trajectories of the static two atoms can be described as


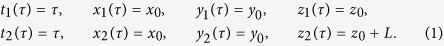


Here we note (*t*_*i*_, *x*_i_, *y*_i_, *z*_i_), (*i* = 1, 2) are the time-space coordinates of the two atoms. At first, let us treat the atoms as closed quantum systems. So the Hamiltonian of the two atoms can be written as [Fig f1]


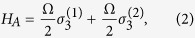


where 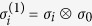, 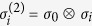, with *σ*_*i*_ (*i* = 1, 2, 3) being the Pauli matrices and *σ*_0_ being the 2 × 2 unit matrix. We assume the two atoms have the same energy level spacing Ω. For time *τ*, a third atom, which carries the information of factor *ϕ* was sent to position P. We assume the state of the third atom is


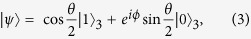


At this time, the initial two atoms remain in state 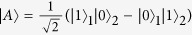. So the total state of the three atoms is the product state of |*ψ*〉 and |*A*〉. Since we want to teleport the information of factor *ϕ* from position P to position Q, we can perform the Bell measurement on the two atoms at position P. According to the result of the measurement, the atomic state at position Q becomes one of the four states 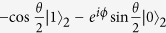, 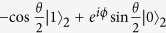, 
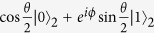
, 
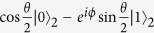
. Here we use QFI to measure the information transmission of factor *ϕ* from position P to position Q. For a two-level system, the state of the system can be expressed in the Bloch sphere representation as


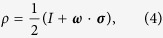


where 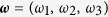 is the Bloch vector and 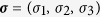 denotes the Pauli matrices. As a result, the QFI of factor *X* can be expressed in a simple form^24^


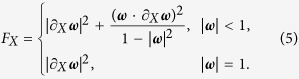


So the QFI of factor *ϕ* for each of the four states at position Q can be calculated and the QFI for each state has the same result


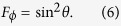


The result is also same as the QFI of the third atomic state |*ψ*〉 before the teleportation. The QFI of factor *ϕ* has been absolutely transmitted from position P to position Q.

However, in reality, every quantum system is open quantum system and the vacuum fluctuations are the unavoidable environment. So the pair of entangled atoms at position P and position Q are also affected by the vacuum fluctuations. As a result, for time *τ*, the two atoms will not remain in state 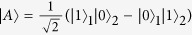. For simplicity, we consider the two-atom system interacting with a bath of fluctuating scalar fields in the Minkowski vacuum. The total Hamiltonian of such a system can then be written as


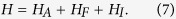


Here *H*_*A*_ is the Hamiltonian of the two atoms as we has discussed before. *H*_*F*_ denotes the Hamiltonian of the scalar fields and the interaction Hamiltonian *H*_*I*_ is taken in analogy to the electric dipole interaction in the weak coupling limit as^25^


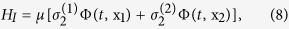


with *μ* denoting the coupling constant and Φ denotes the strength of scalar field.

We use 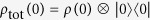 to denote the initial state, which means the atoms and the environment are uncorrelated at the beginning as supposed. Here |0〉 is the Minkowski vacuum state of the scalar fields, and 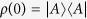 is the initial state of the two-atom system. In the weak-coupling limit, the reduced dynamic of the two-atom system takes the Kossakowski-Lindblad form^26^,^27^,^28^


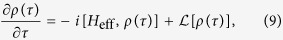


with


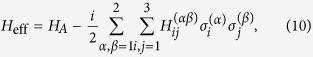


and


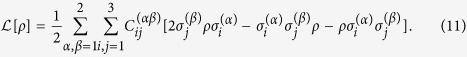


Here 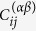 and 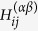 are determined by the Fourier and Hilbert transforms, 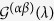 and 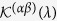 with^26^,^27^,^28^,^29^,^30^


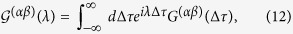



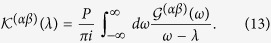


Here


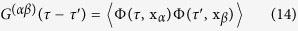


denote the field correlation functions and *P* denotes the principal value.

Then 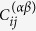 can be written explicitly as


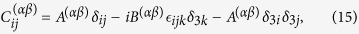


where


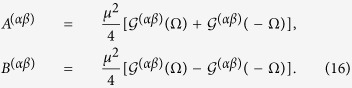


Replacing 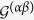 with 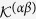 in the above equations, 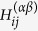 can be obtained.

The Wightman function of massless scalar fields in the Minkowski vacuum takes the form


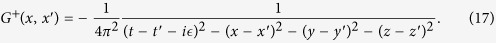


Allowing for the trajectories (1), the correlation functions can be written as


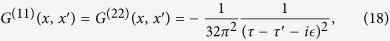



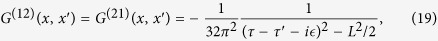


where 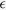 denotes a small constant with 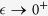. The Fourier transforms of the above correlation functions are


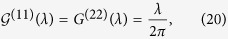



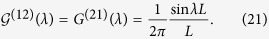


Then the coefficients of the dissipator in the master equation can be obtained directly as


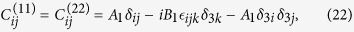



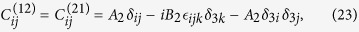


where


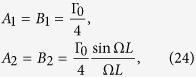


with 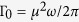 being the spontaneous emission rate.

Using the coupled basis 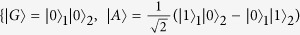, 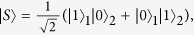

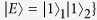
, then a set of equations being used to describe the time evolution of the populations of some matrix elements, which are decoupled from other matrix elements, can be obtained as^29^,^30^


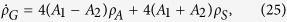



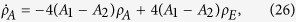



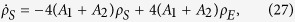



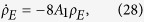


where *ρ*_*I*_ = 〈*I*|*ρ*|*I*〉, *I* ∈ {*G, A, S, E*}. Since *ρ*_*G*_ + *ρ*_*A*_ + *ρ*_*S*_ + *ρ*_*E*_ = 1, only three of the above equations are independent.

In the present case, *ρ*_*A*_(0) = 1, *ρ*_*G*_(0) = *ρ*_*S*_(0) = *ρ*_*E*_(0) = 0, so we have


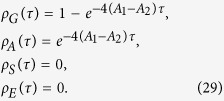


As a result, the state of initially entangled two-atom system at time *τ* becomes 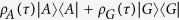 and the total state of the three atoms before teleportation becomes the product state of |*ψ*〉 〈*ψ*| and 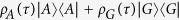. Now let us perform the Bell measurement at position P. Then, the teleported state becomes one of the four states


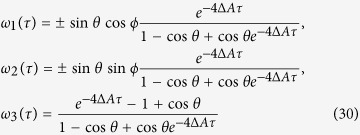


and


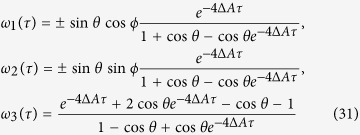


with Δ*A* = *A*_1_ − *A*_2_. So the teleported QFI of parameter *ϕ* becomes


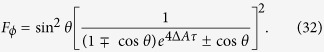


The results show that causing by the interaction between vacuum fluctuations and the pair of atoms, the teleported QFI of parameter *ϕ* decreases with time and the decay rates depend on


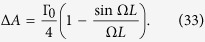


When the wavelength of the atoms is much smaller than the distance between position P and Q (Ω*L* → ∞), 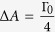. So the decay rates of the QFI depend on the spontaneous emission rate of the atoms. However, when the wavelength of the atoms is much larger than the distance between position P and Q (Ω*L* → 0), Δ*A* → 0. The atoms interact with the field modes in a collective and coherent way and the teleported QFI of parameter *ϕ* remains sin^2^ *θ*, which means the QFI of factor *ϕ* has been absolutely transmitted from position P to position Q in the existence of vacuum fluctuations.

For *θ* = *π*/2, the teleported QFI for each measurement has the same result *F*_*ϕ*_ = *e*^−8Δ*Aτ*^. We find that the teleported QFI decreases exponentially and the decay rate becomes 8Δ*A*. We plot Δ*A* (in the unit of 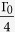) as the function of Ω*L*. We find that with the increase of atomic distance in comparing with the wavelength of the atom, the decay rate increases oscillatorily.

## Conclusion

In conclusion, we have studied the teleported QFI of phase parameter of atomic state by using two initially entangled static two-level atoms, which coupled to a bath of fluctuating vacuum scalar field in two different places. Our results show that for given time, the teleported QFI is determined by the wavelength of the atoms as well as the distance of teleportation. When the wavelength of the atoms is much smaller than the teleportation distance, the teleported information decays with time and the decay rates are determined by the spontaneous emission rate of the atoms. However, when the wavelength of the atoms is much larger than the teleportation distance, the QFI of the phase parameter of atomic state remains sin^2^ *θ* and the information of the phase parameter of atomic state has been absolutely transmitted.

## Additional Information

**How to cite this article**: Jin, Y. The effects of vacuum fluctuations on teleportation of quantum Fisher information. *Sci. Rep.* **7**, 40193; doi: 10.1038/srep40193 (2017).

**Publisher's note:** Springer Nature remains neutral with regard to jurisdictional claims in published maps and institutional affiliations.

## Figures and Tables

**Figure 1 f1:**
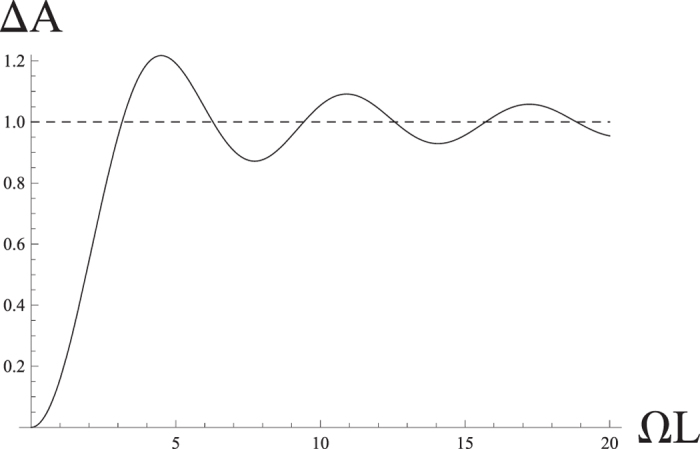
The decay rate of QFI as function of atomic distance.
